# Supplementary material for: Generation of Lasso Peptide-Based ClpP Binders
Source: Int J Mol Sci. 2021 Dec 31;23(1):465. doi: 10.3390/ijms23010465 (PMC8745299; doi:10.3390/ijms23010465)
Supplement: Supplementary file 1 [file ijms-23-00465-s001.zip › ijms-1519807-supplementary.pdf]

SUPPORTING INFORMATION

Generation of Lasso Peptide-based ClpP Binders

Imran T. Malik,[1] Julian D. Hegemann,[2,*] and Heike Brötz-Oesterhelt[1,3,*]

[1] Department of Microbial Bioactive Compounds, Interfaculty Institute of Microbiology and Infection Medicine, University of Tübingen, Auf der Morgenstelle 28, 72076 Tübingen, Germany.

[2] Helmholtz Institute for Pharmaceutical Research Saarland (HIPS), Helmholtz Centre for Infection Research (HZI), Saarland University Campus, 66123 Saarbrücken, Germany.

[3] Cluster of excellence "Controlling Microbes to Fight Infection"

* Correspondence: jdhegemann@googlemail.com (J. D. H.); heike.broetz-oesterhelt@uni-tuebingen.de (H. B.-O.)

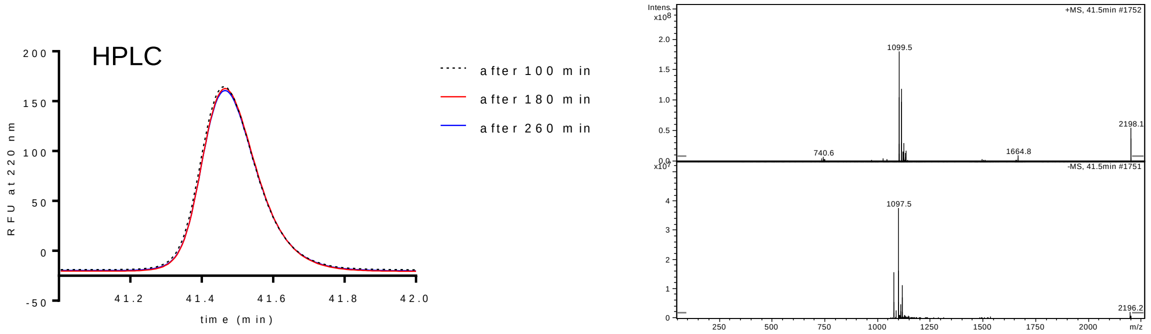

Figure S1. LC-MS analysis of SaClpP with 12IGF. SaClpP (1µM) and 12IGF (46 µM) were incubated under *in vitro* assay conditions for up to 260 min and analyzed via HPLC. No reduction in 12IGF amounts could be observed. Identity of 12IGF (2196 kDa) was confirmed by mass spectrometry at a retention volume of 41.5 ml.

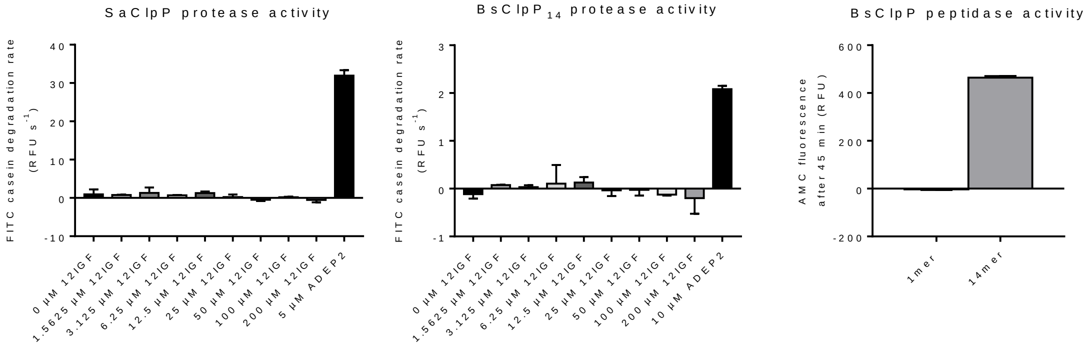

Figure S2. FITC-casein degradation by SaClpP and tetradecameric BsClpP (tetradecamer conditions applied as described in the main text) at different 12IGF concentrations and 5 µM or 10 µM ADEP2 as positive controls, respectively. No activation of casein degradation by 12IGF was detected. *Right panel*, the tetradecameric state of BsClpP was confirmed by stand-alone peptide hydrolysis (i. e. without addition of ADEP2 or 12IGF) with a monomeric preparation as a negative control.

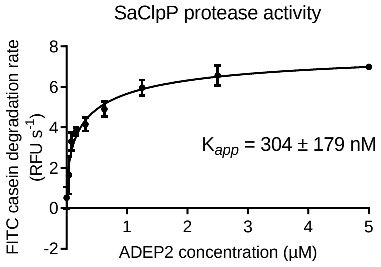

Figure S3. FITC-casein degradation by 100 nM of SaClpP at different ADEP2 concentrations.
